# Post-mortem inference of the human hippocampal connectivity and microstructure using ultra-high field diffusion MRI at 11.7 T

**DOI:** 10.1007/s00429-018-1617-1

**Published:** 2018-01-31

**Authors:** Justine Beaujoin, Nicola Palomero-Gallagher, Fawzi Boumezbeur, Markus Axer, Jeremy Bernard, Fabrice Poupon, Daniel Schmitz, Jean-François Mangin, Cyril Poupon

**Affiliations:** 1 CEA NeuroSpin/UNIRS, Gif-sur-Yvette, France; 2 Université Paris-Saclay, Orsay, France; 3 France Life Imaging, Orsay, France; 4 Forschungszentrum Jülich, INM-1, Jülich, Germany; 5 Department of Psychiatry, Psychotherapy and Psychosomatics, Medical Faculty, RWTH Aachen, Aachen, Germany; 6 CEA NeuroSpin/UNATI, Gif-sur-Yvette, France; 7 CATI Neuroimaging Platform, http://catineuroimaging.com

**Keywords:** Diffusion MRI, Human hippocampus, Structural connectivity, Neurite density, Microstructure imaging

## Abstract

The human hippocampus plays a key role in memory management and is one of the first structures affected by Alzheimer’s disease. Ultra-high magnetic resonance imaging provides access to its inner structure in vivo. However, gradient limitations on clinical systems hinder access to its inner connectivity and microstructure. A major target of this paper is the demonstration of diffusion MRI potential, using ultra-high field (11.7 T) and strong gradients (750 mT/m), to reveal the extra- and intra-hippocampal connectivity in addition to its microstructure. To this purpose, a multiple-shell diffusion-weighted acquisition protocol was developed to reach an ultra-high spatio-angular resolution with a good signal-to-noise ratio. The MRI data set was analyzed using analytical Q-Ball Imaging, Diffusion Tensor Imaging (DTI), and Neurite Orientation Dispersion and Density Imaging models. High Angular Resolution Diffusion Imaging estimates allowed us to obtain an accurate tractography resolving more complex fiber architecture than DTI models, and subsequently provided a map of the cross-regional connectivity. The neurite density was akin to that found in the histological literature, revealing the three hippocampal layers. Moreover, a gradient of connectivity and neurite density was observed between the anterior and the posterior part of the hippocampus. These results demonstrate that ex vivo ultra-high field/ultra-high gradients diffusion-weighted MRI allows the mapping of the inner connectivity of the human hippocampus, its microstructure, and to accurately reconstruct elements of the polysynaptic intra-hippocampal pathway using fiber tractography techniques at very high spatial/angular resolutions.

**Electronic supplementary material:**

The online version of this article (10.1007/s00429-018-1617-1) contains supplementary material, which is available to authorized users.

## Introduction

The human hippocampal formation plays a critical role in learning and memory. Its regions appear to be specialized for preferential functions, such as the specific involvement of the dentate gyrus (DG) and the Cornu Ammonis (CA) subfield 3 in pattern separation and completion, respectively, and that of CA2 in social memory (Leutgeb et al. 2007; Hitti and Siegelbaum 2014). Furthermore, the structure and connectivity of hippocampal regions and layers are known to be selectively affected by multiple neurological disorders such as Alzheimer’s disease or temporal lobe epilepsy, as well as by the normal process of aging (Zhou et al. 2008; Wang 2006; Dinkelacker et al. 2015; Coras 2014; Prull et al. 200; Wilson et al. 2006). Although anterior–posterior (ventral–dorsal in rodents) functional differences within the hippocampal formation have been reported in humans and experimental animals (Fanselow and Dong 2010; Poppenk et al. 2013), detailed anatomical studies are still not available in humans. The complexity of the hippocampus makes it one of the most mysterious regions in the central nervous system and also one of the most extensively studied. The boundaries between different hippocampal subfields have been described in the neuroanatomy literature using cytoarchitectonic features that require histological staining and microscopic resolution to visualize (Gloor 1997; Amaral and Insausti 1990; Duvernoy 2005), but there are still discrepancies concerning whether it makes sense to separate some regions or not.

Few in vivo imaging techniques are available to investigate the human hippocampus. Magnetic resonance imaging (MRI), and more precisely anatomical MRI (aMRI) and diffusion MRI (dMRI), remain the key modalities used nowadays. Studies using aMRI were first employed to segment the hippocampus and enable volumetric analysis to define early markers of Alzheimer’s disease or depression (Jack et al. 1992; Videbech and Ravnkilde 2004; Boutet 2014). Several studies have also been conducted using diffusion tensor imaging (DTI) on clinical MRI systems to investigate the connectivity of the human hippocampus to better understand the anatomo-functional mapping of the limbic system (Adnan et al. 2015; Dinkelacker et al. 2015; Zeineh et al. 2012). However, clinical MRI systems have inherent limitations that prevent one from reaching ultra-high spatial and angular resolutions, mainly due to the characteristics of the available gradient coils. The recent development of the Connectome gradient, able to provide 300 mT/m, could offer an alternative, but it is still limited in spatial resolution (1.25 mm) due to the static field being kept moderate (3T) to prevent the strong mechanical constraints that would occur at ultra-high field (McNab 2013; Setsompop 2013).

Alternatively, ex vivo MR imaging can be performed using ultra-high field preclinical scanners. In addition to the ultra-high static field, these systems can be equipped with very strong gradients that can reach 1000 mT/m. The *b*-values can thus exceed 10,000 $\hbox {s}/\hbox {mm}^{2}$, diffusion times can be short enough to reach short diffusion time regimes, and diffusion gradient pulses become closer to Dirac shapes. Finally, contrary to in vivo scans that cannot exceed a couple of hours, ex vivo imaging does not suffer from such a limitation. Specifically, ex vivo studies have thus been carried out with medial temporal lobe (MTL) samples containing the hippocampus (Shepherd et al. 2007; Augustinack 2010; Coras 2014; Colon-Perez 2015; Modo et al. 2016), where most of the authors carried out DTI-based tractography to map some of the larger connections of the hippocampus, such as the perforant pathway (Augustinack 2010; Coras 2014). Although they remain landmark studies, these studies rely on a model known to present strong limitations. First, it cannot model multiple fiber populations within a voxel, like bundle crossings or kissings, which is a weakness especially in the case of the hippocampus, because it contains multiple fiber crossings reflecting its complex circuitry. Second, diffusion tensor features like fractional anisotropy (FA) and mean diffusivity (MD) are inherently non-specific, and a reduction in their value can be associated with different types of microstructural changes. For example, a reduction in FA can be due to demyelination, edema, increased neurite dispersion, or other microstructural changes (Takahashi 2002; Beaulieu 2009; De Santis et al. 2014).

To model multiple fiber populations within a voxel, numerous reconstruction techniques have been developed during the last decades. Regarding modeling of regions with a complex fiber architecture, High Angular Resolution Diffusion Imaging (HARDI) is probably the most widely adopted (see Tournier et al. (2011) for a review of such models). HARDI models produce maps of orientation distribution functions (ODF), the peaks of which characterize the diffusion displacement profile. Since the hippocampus has a very complex fiber architecture with crossings, kissings, and splittings of fibers, it becomes mandatory to use HARDI models to robustly infer its structural connectivity using tractography. To our knowledge, HARDI models have only been used once on a human hippocampus, by Colon-Perez (2015), and not to study the human hippocampal connectivity in its entirety.

Several reconstruction techniques have recently emerged in diffusion MRI to characterize the tissue microstructure, yielding new applications aiming at doing virtual biopsy, and known as diffusion MR microscopy. They rely on the development of multi-compartmental models that estimate, for each voxel, the fraction of each compartments and its key characteristics (e.g., density or dimension). The first established technique relying on the Composite Hindered And Restricted ModEl of Diffusion (CHARMED) introduced by Assaf and Basser (2005) assumed a diffusion attenuation resulting from three compartments. This model laid the foundations of the two next techniques aiming at providing estimates of the axon diameter and density, i.e., AxCaliber (Assaf et al. 2008) and ActiveAx (Alexander et al. 2010), as well as their improvements (Zhang et al. 2011; De Santis et al. 2016). More recently, the Neurite Orientation Dispersion and Density Imaging (NODDI) reconstruction technique was introduced (Zhang et al. 2012) to quantify axon and dendrite densities (collectively known as neurites). These parameters have been shown to provide more specific characteristics of brain tissue microstructure than the quantitative parameters derived from the DTI model.

For the first time, we demonstrate on a human medial temporal lobe sample that ex vivo ultra-high field with strong gradients MRI at 11.7 T and 780 mT/m gives the opportunity to map not only the complex anatomy of the hippocampus, but also its inner connectivity and its organization at the mesoscopic scale. First, we investigate the use of combined anatomical and diffusion MRI to segment the inner structures of the hippocampus and the adjacent entorhinal cortex. Second, we demonstrate that HARDI allows one to accurately reconstruct elements of the polysynaptic pathway of the hippocampal formation. Third, we show that diffusion MR microscopy is a powerful technique, that gives access to insights about cell populations of the hippocampal tissues by revealing the laminar structure of the cornu ammonis (CA).

## Materials and methods

### Tissue sample and container

The study was carried out on a post-mortem right temporal lobe from an 87-year-old male with no abnormal neuropathology findings obtained from the donor program of the Institute of Anatomy, Rostock, Germany. The specimen measured approximately $38\times 50\times 55\ \hbox {mm}^{3}$, and contained the whole hippocampal formation and some of its surrounding structures. It was fixed with $4\%$ formalin buffer 36 h after death and stored 38 months until further processing.

Prior to MRI acquisition, the sample was transferred for 1 week to a 0.1 M phosphate-buffered saline solution at $4\,^{\circ }\hbox {C}$ to be rehydrated. Since acquisitions take place at room temperature (approximately $20\,^{\circ }\hbox {C}$), the specimen was placed into the imaging container 4–5 h prior to scanning and transferred to the magnet room to stabilize its temperature to $20\,^{\circ }\hbox {C}$. This process is required to avoid effects related to temperature variations that would induce modifications of the local $T_{2}$ relaxation time and the local apparent diffusion coefficient (ADC) up to a factor of 1.5–2 between 4 and $20\,^{\circ }\hbox {C}$ (Thelwall et al. 2006). Note that the ADC of this post-mortem sample being scanned at $20\,^{\circ }\hbox {C}$, instead of $37\,^{\circ }\hbox {C}$ as for in vivo imaging, was already reduced by a factor of about 2. Furthermore, to avoid drying of the tissue during the experiment and to reduce imaging artifacts, the sample was immersed in a proton-free fluid, Fluorinert (FC-40, 3M Company, USA). This fluid does not provide any NMR signal and shares a similar susceptibility coefficient to the one of brain tissues, enabling one to avoid the induction of static magnetic field variations close to the interfaces between air and tissue that would induce local geometrical and intensity distortions. A dedicated container was manufactured to exactly fit the inner diameter of the MR coil antenna with a specific design aimed to prevent the formation of air bubbles that would be responsible for severe susceptibility-induced imaging artifacts. The suspension of the sample within its container is guaranteed by a plastic funnel that does not induce any MR signal and avoids motion artifacts during the commutation of the strong diffusion gradients.

### MRI hardware

All the acquisitions were performed on a preclinical 11.7 T Bruker MRI system (BioSpec 117/16 USR Bruker MRI, Ettlingen, Germany) equipped with strong gradients (maximum gradient $\hbox {magnitude} = 780\,\hbox {mT}/\hbox {m}$, slew-$\hbox {rate} = 9660\ \hbox {T}/\hbox {m}/\hbox {s}$) using a 60 mm transmit/receive volume coil. Although surface coils are known to provide better SNRs than volume coils, the 60 mm volume coil was preferred, because it corresponded to the best trade-off between the field-of-view (FOV) coverage and the dimensions of the sample. Furthermore, it enabled preservation of a relative homogeneity of the signal through its entire FOV.

### Determination of the magnetic and diffusion properties of the sample

It was mandatory to calibrate the distributions of the magnetic and diffusion properties of the hippocampus sample to adequately tune the target diffusion-weighted multiple-shell imaging protocol required to apply the NODDI model. To this aim, a calibration MRI protocol was established including a series of experiments to infer the histograms of the $T_{2}$ transverse relaxation time and the mean diffusivity *D*. Their analysis helps to define the maximum value of the diffusion sensitization b to be used. For a conventional Pulsed-Gradient-Spin-Echo (PGSE) diffusion-weighted imaging sequence, $\hbox {b} =(\mathbf |G| \gamma \delta )^{2}(\varDelta -\delta /3)=\mathbf |q| ^{2}\tau$ with the approximation of rectangular gradients, with **G** the applied diffusion gradient vector, $\gamma$ the nuclear gyromagnetic ratio for water protons, $\delta$ the duration time of gradient pulses, $\varDelta$ the time between two pulses, and $\tau$ the diffusion time.

To reach high *b*-values, one can increase the gradient strength **G**, the gradient width $\delta$, or the separation time between the two gradient pulses $\varDelta$. However, to keep the gradient pulses close enough to Dirac pulses, thus preserving a Fourier relationship between the diffusion propagator and the diffusion NMR signal with respect to the *q* wavevector, $\delta$ has to be kept at its minimum possible value, 4.3 ms in our case. Consequently, either $\varDelta$ or *G* have to be raised to increase the diffusion sensitization. The side effect of increasing $\varDelta$ is a net increase of the echo time $T_\text {E}$. However, the PGSE sequence yields an NRM signal that integrates not only an exponential diffusion decay $e^{-bD}$, but also a further exponential decay $e^{-T_\text {E}/T_{2}}$ linked to the $T_{2}$ transverse relaxation inherited from the spin-echo scheme present in a PGSE sequence.

An estimation of *D* and $T_{2}$ was carried out to determine the range of usable echo time $T_\text {E}$ and diffusion sensitization *b*, to avoid excessive signal loss.

#### Histogram of the transverse relaxation time $T_{2}$

Fixed tissues generally suffer from a significant reduction of their transverse relaxation time drastically reducing the time window available to acquire the signal with a good signal-to-noise ratio (SNR) (Pfefferbaum et al. 2004). To map the transverse relaxation time at each voxel of our sample, a standard multi-spin-multi-echo (MSME) pulse sequence (Meiboom and Gill 1958) was used. In total, 12 echoes were collected corresponding to 12 echo times linearly spaced between 6.4 and 76.8 ms. Imaging parameters for this sequence were: isotropic spatial resolution of $300\,\upmu \hbox {m}$, 12 averages, $\hbox {TR} = 16,000\,\hbox {ms}$, and a total scan duration of 10 h 14 min. Collected MSME data were then used to fit the log-linear model corresponding to the $T_{2}$-decay with a Levenberg–Marquardt algorithm carefully initialized to get robust estimates. The histogram of the $T_{2}$ quantitative values computed from the voxels included in a precomputed mask of the sample and depicts two main modes of $T_{2}$. The mode corresponding to the lower value is identified as the white matter $T_{2}$ ($T_{2w}$ $\approx$ 36.3 ms), and that corresponding to the higher value is identified as grey matter $T_{2}$ ($T_{2g}$ $\approx$ 46.4 ms): transverse relaxation time is lower where fibers are more concentrated, i.e., in the white matter. As the hippocampus is mainly composed of grey matter, we assumed its $T_{2}$ value closer to 46 ms.

#### Histogram of the mean diffusivity *D*

Post-mortem tissues depict diffusion coefficients significantly lower (two to five times lower) than in vivo values. Consequently, probing the anisotropy of the diffusion process requires the use of inversely proportional higher *b* values, to obtain a comparable diffusion contrast to in vivo images (D’Arceuil et al. 2007). A histogram of the mean diffusivity *D* was inferred using the DTI model from a diffusion-weighted data set acquired with a single-shell sampling of the *q*-space at $b=4500\, \hbox {s}/\hbox {mm}^2$ along 60 uniformly distributed diffusion directions, $\hbox {TR}/\hbox {TE} = 9000/24.2\,\hbox {ms}$, $\varDelta /\delta = 14.4/4.3\,\hbox {ms}$, matrix size: $192 \times 192 \times 176$, and an isotropic resolution of $300\,\upmu$m. The distribution indicates a mean diffusivity of $0.16 \times 10^{-3}\ \hbox {mm}^{2}/\hbox {s}$.

#### Determination of maximum $T_\text {E}$ and *b* values

To prevent an excessive loss of signal, a lower acceptable limit of the product $e^{\frac{-\text{TE}}{T_{2}}}\ e^{-bD}$ was set to 0.05, equally distributed over the two signal decays:

1 $$\begin{aligned} \left\{ \begin{array}{ccc} e^{\frac{-\text {TE}_{\text {max}}}{T_{2}}} &{} \ge &{} \sqrt{0.05}\\ e^{-b_{\text {max}}D} &{} \ge &{} \sqrt{0.05},\\ \end{array}\right. \end{aligned}$$

and hence

2 $$\begin{aligned} \left\{ \begin{array}{cccc} \text {TE}_{\text {max}} &{} \le &{} 69 &{}\\ e^{-b_{\text {max}}D} &{} \le &{} 9361 &{} \hbox {s}/\hbox {mm}^{2}.\\ \end{array}\right. \end{aligned}$$

In practice, $TE_{\text {max}}$ was set to 59.1ms, which enabled a $b_{\text {max}}$ of 10,000 $\hbox {s}/\hbox {mm}^{2}$.

### Imaging protocol

The imaging protocol included anatomical and diffusion scans. The anatomical scan was tuned to reach a very high spatial resolution to perform accurate manual segmentation of the hippocampal subfields and of the entorhinal cortex.

#### Anatomical scan

The anatomical image was acquired using a standard $T_{2}$-weighted spin-echo sequence with isotropic spatial resolution of $200\,\upmu \hbox {m}$, matrix $268 \times 268$; 256 slices, thickness $200\,\upmu \hbox {m}$, $\hbox {TR}/\hbox {TE}$ = 12,000$/26.5\,\hbox {ms}$, 15 averages, and a total scan time of 10 h 46 min.

#### Diffusion scan

Diffusion data were acquired using a conventional PGSE sequence. The protocol included a first single-shell high angular resolution diffusion imaging (HARDI) data set used to infer the structural connectivity of the human hippocampal formation, to which a multiple-shell hybrid diffusion imaging (HYDI) data set was added to infer quantitative microstructural features using diffusion MR microscopy.

The structural connectivity was established from the HARDI data set collected at $\hbox {b} = 4500\ \hbox {s}/\hbox {mm}^{2}$ along 500 directions uniformly distributed over a sphere. It was split into 15 blocks of 32 directions and one of 16 because of the memory limitation of the system. For each block, 6 ${b} = 0$ images were acquired. Scanning parameters were: isotropic spatial resolution of $300\,\upmu \hbox {m}$; $\hbox {TR}/\hbox {TE} = 9000/24.2\,\hbox {ms}$; $\varDelta /\delta = 14.4/4.3\,\hbox {ms}$; matrix size: $192 \times 192$; 176 slices; and a total scan time of 8 days and 18 h. The *b* value was calibrated taking into account the reduction of the average diffusivity from $D = 0.7 \times 10^{-3}\,\hbox {mm}^2/\hbox {s}$ in vivo to $D = 0.16 \times 10^{-3}\,\hbox {mm}^2/\hbox {s}$ in our case, to compensate for the loss of contrast due to the observed reduction factor. As mentioned earlier, the data set was used first to perform tractography, but also to map microstructural features when merged with the next HYDI data set.

In addition to the HARDI data set, a further multiple-shell HYDI data set was acquired for 3 different shells corresponding to 3 different diffusion sensitization at $\hbox {b} = 4500\, \hbox {s}/\hbox {mm}^{2}$, $\hbox {b} = 7500\, \hbox {s}/\hbox {mm}^{2}$ and $\hbox {b}$ = 10,000 $\hbox {s}/\hbox {mm}^{2}$. The choice of the *b*-values was carried out based on the usual sensitizations taken for NODDI or ActiveAx models with a scaling factor of around 3 applied to account for the attenuation of the diffusivities observed ex vivo. For each shell, 60 diffusion-weighted volumes were acquired along 60 uniformly distributed diffusion directions. The acquisition was divided into two blocks of 30 directions each. Each block lasted 12 h 57 min, giving a total of 3 days and 7 h. The parameters were tuned as listed in Table 1. It is important at this step to note that the three shells were acquired with linearly increasing separation times $\varDelta$ of 14.4, 30.0, and 45.0 ms while keeping the gradient pulse width to $\delta = 4.3\,\hbox {ms}$, to vary the diffusion time. This choice was motivated by the willingness to be able to use alternative models to NODDI in the future, such as ActiveAx, which also requires sampling of the diffusion time. A further specificity of the HYDI protocol was to use the minimum echo time for each shell and not to impose the largest of the three minimum echo times. As a consequence, the data stemming from each shell have to be preprocessed to remove the $T_{2}$-decay dependency, which is possible thanks to the quantitative $T_{2}$ calibration performed previously to characterize the magnetic and diffusion properties of the hippocampus sample.

**Table 1:** Parameters for the NODDI data set

b ($\hbox {s}/\hbox {mm}^{2}$)	$N_{\text {dir}}$	TR (ms)	TE (ms)	$\varDelta$ (ms)	G (mT/m)
4500	30	9000	24.2	14.4	684
4500	30	9000	24.2	14.4	684
7500	30	9000	39.8	30.0	595
7500	30	9000	39.8	30.0	595
10,000	30	10,315	54.8	45.0	556
10,000	30	10,315	54.8	45.0	556

### Delineation of the inner and surrounding structure of the hippocampus

In-house developed software (by FP), PtkVoi, was used to trace the hippocampal subfields and surrounding structures. It was performed manually by two independent experts from the $T_{2}$-weighted anatomical scan, using anatomical landmarks and following the different strategies prescribed and detailed in the literature (Insausti 1998; Duvernoy 2005; Wisse et al. 2012; Insausti and Amaral 2012; Boutet 2014).

Segmentations were traced in the coronal plane and the three-dimensional consistency was ensured by checking the axial and sagittal planes, as well as the 3D shapes of the delineated structures with respect to their known morphology. Considering the main intra-hippocampal circuits, we endeavored to identify: the entorhinal cortex, the dentate gyrus (including the hilus), CA2/CA3, CA1, the alveus, the fimbria, and the subicular complex.

#### Inner structures of the hippocampus

The number of inner segmented structures of the hippocampus resulted from a trade-off between the feasibility to identify their boundaries on the high-resolution anatomical MRI data (thus depending on their contrast to noise ratio), and the target regions involved in the circuits of interest. The segmentation process used a coarse to fine strategy involving a first step in which the hippocampal head, body, and tail were identified, followed by a second step to delineate regions and/or layers.

#### Coarse scale: segmentation of head, body, and tail

The segregation of the hippocampus in three parts was based on the protocol presented in Boutet (2014). The very anterior limit is not present in our sample, because the head was partly cut. The most anterior part of the body corresponds to the first coronal slice where the median part of the uncus is no longer visible. The most posterior coronal slice of the body was identified at the level of the enlargement of the fimbria and the loss of the specific C-shape of the body.

#### Fine scale: segmentation of subregions

**Fig. 1:**
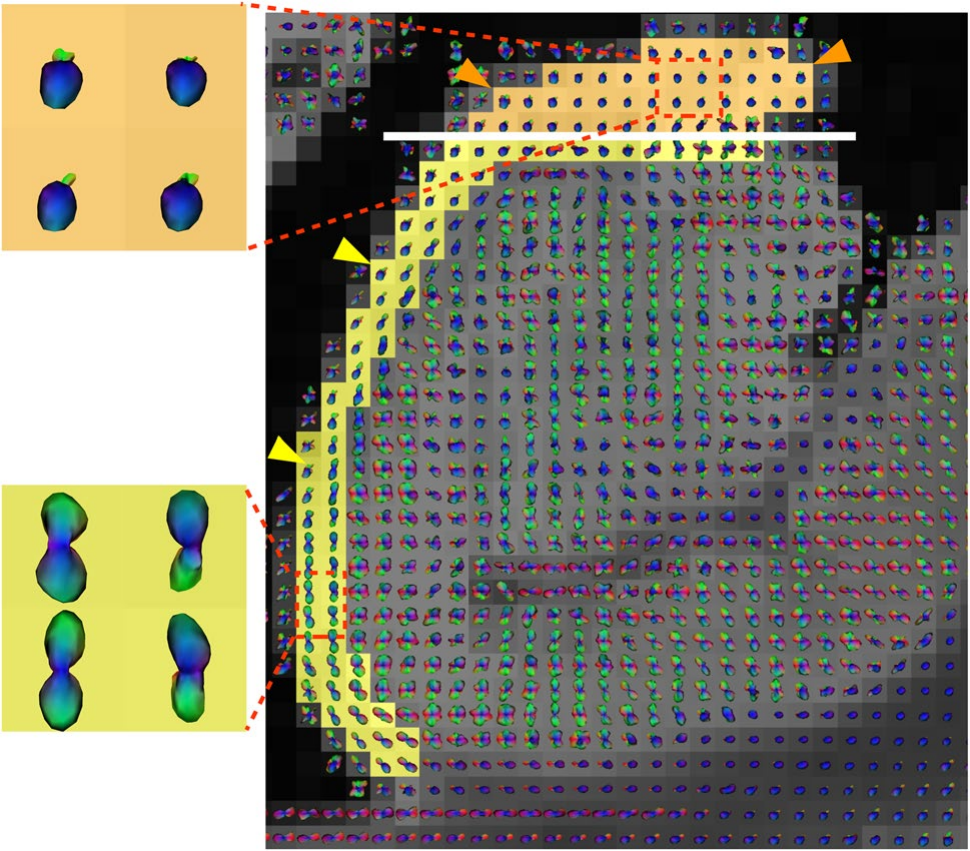
Close view of the shift in fiber orientation between alveus and fimbria. Yellow arrowheads highlight typical inferior–superior orientation of ODFs in the alveus (fibers in the coronal plane, as shown on upper left). Orange arrowheads highlight typical anterior–posterior orientation of ODFs in the fimbria (fibers in the sagittal plane, as shown on upper left). White line shows the boundary between alveus and fimbria, defined within the transition zone corresponding to the shift in the fiber orientation

In a second step, we segmented the following structures (3):

1. Alveus and fimbria. These two structures belong to the white matter and are visible in the anatomical $T_{2}$-weighted MRI data by the hypointense contrast at the level of the outer boundary of the hippocampus. This specific contrast was exploited to guide their delineation. In addition, the change in orientation of the underlying fibers between the alveus (oriented mainly parallel to the coronal plane) and the fimbria (oriented mainly parallel to the sagittal plane) can be easily identified in diffusion ODF fields, thus allowing the identification of the boundary between the alveus and the fimbria. The lateral boundary of the fimbria was set at the end of the fibers orientation shift zone, where the fibers are entirely contained in the sagittal plane (white line in Fig. 1). The inferior lateral boundary of the alveus was set at the junction with the collateral eminence of the lateral ventricle. The separation between the alveus and the fimbria was only possible in the body and the tail of the hippocampus. At the level of the hippocampal head, all white matter was associated with the alveus.
2. The lacunosum-molecular layer of the CA1–CA3 regions. This zone can easily be identified in the anatomical $T_{2}$-weighted MRI scan as a dark line in the CA region of the hippocampus, corresponding to an area of very low concentration of neural bodies (which are mainly located in the pyramidal layer).
3. The pyramidal and radiatum layers of the CA1 region. This region is the thickest Ammon’s field, while CA2 is the thinnest, thus easily enabling definition of the boundary between both portions of the hippocampus. The border with the subicular complex could often be identified based on differences in grey values, since the CA1 region appeared brighter when compared to the adjacent subicular component. In the tail region of the hippocampus, where differences in grey values were only very subtle, we applied the method suggested by Boutet (2014), which consists of tracing the largest diameter of the hilum and a perpendicular line passing by the medium of the diameter that corresponds to the target boundary.
4. The pyramidal layer and radiatum of the CA2 and CA3 regions. These two regions were merged into a single ROI (CA2/CA3), because the boundary between them is almost impossible to identify in MR images. Furthermore, not only CA3 neurons project to the CA1 region, but also CA2 pyramids make strong excitatory synaptic contacts with CA1 neurons (Chevaleyre and Siegelbaum 2010). The limit with the dentate gyrus was defined by the end of the ribbon-like aspect of the Ammon’s horn (Boutet 2014).
5. The dentate gyrus. Its boundaries were defined by the other structures already segmented and the cisterna ambiens enlarging the cerebro-spinal fluid-filled space lateral to the cerebral crus.
6. The subicular complex. The lateral limit of this region was identified based on differences in cortical thickness, as described by Wisse et al. (2012). Thus, the border was defined by drawing a line between the most medial part of the grey matter and the most medial part of the white matter of the temporal stem.
7. The entorhinal cortex. Its lateral limit is slightly upstream of the collateral sulcus, itself located under the collateral eminence. In Wisse et al. (2012), the posterior border of the entorhinal cortex was set 0.7 mm beyond the hippocampal head which corresponds to four slices for our spatial resolution.

### Connectivity and microstructure mapping

#### Preprocessing

As mentioned earlier, the diffusion-weighted imaging protocol included the acquisition of a multiple-shell HYDI data set at three different *b* values (4500, 7500, 10,000 $\hbox {s}/\hbox {mm}^{2}$) with minimum echo times set for each individual shell. This choice was motivated by the optimization of the SNR per shell, but it requires to correct for the $T_{2}$-weighting equal to $e^{\frac{-\rm {TE}}{T_{2}}}$ that differs from one shell to another. To this aim, a correction was applied to the two larger shells at 7500 and 10,000 $\hbox {s}/\hbox {mm}^{2}$ that consisted of multiplying voxelwise all the diffusion-weighted images by the compensating factors $e{^{\frac{\varDelta {\text{TE }}}{ T_{2} (v) }}}$ for each voxel *v*. This resulted in an intensity comparable to the one obtained with an acquisition done with the TE used at ${b} = 4500\,\hbox {s}/\rm {mm}^{2}$ for all the shells.

The conventional PGSE sequence is not sensitive to field inhomogeneities in comparison with its corresponding echoplanar version, such that the collected DW data are free from distortions induced by eddy currents or susceptibility effects. Consequently, there is no need to apply any correction. In addition, the measured SNR established to 9.9, 7.6, and 4.2 for the three shells, respectively, corresponding to an increasing *b*-value, are large enough to approximate the Rician noise distribution by a Gaussian distribution, thus allowing the use of conventional mean-square estimators without loss of information. Finally, all the acquisitions were performed during 13 consecutive days avoiding the need to remove the sample from its container, and thus avoiding the presence of any motion between two diffusion-sensitized volumes. Consequently, the remaining transformation exiting between the two anatomical and diffusion data sets turns to be the simple rigid transformation between their two fields of view. This transformation was inferred using the registration tool of the Connectomist toolbox (Duclap et al. 2012; Assaf 2013) based on a mutual information matching criterion optimized using a standard Nelder–Mead simplex algorithm. After registration, the two data sets perfectly matched, allowing to navigate between the structures inferred from the anatomical scans and the connectivity or microstructural quantitative maps inferred from diffusion MRI scans.

#### Choice of the local reconstruction model

Inference of the local orientation distribution function of the diffusion process or of the fiber orientation distribution can be done using two classes of HARDI techniques: modeldependent reconstructions and model-free reconstructions. Among model-dependent reconstructions, that impose a specific impulse response of a fiber bundle to the diffusion process are the Ball and Sticks model of Behrens (2003), the Constrained Spherical Deconvolution of Tournier and Calamante (2007) or the Sharpening Deconvolution Transform of Descoteaux et al. (2009). Among model-free reconstructions, that do not make any assumption about the response of an heterogeneous population to the diffusion process, are the Diffusion Spectrum Imaging model of Wedeen et al. (2000), the analytical Q-ball model of Descoteaux et al. (2007), the Diffusion Orientation Transform of Özarslan et al. (2006), or the latest Simple Harmonic Oscillator-Based Reconstruction and Estimation (SHORE) propagator model of Özarslan et al. (2013).

The current trend is to use spherical deconvolution approaches to obtain sharp fiber orientation distributions resulting from the deconvolution of ODFs using an auto-estimated convolution kernel. While this approach is suitable to reconstruct the connectivity of the entire brain, it cannot be considered for this study of the inner hippocampal connectivity where the complex configuration of fibers does not enable the definition of an adequate convolution kernel: there is no equivalent of the corpus callosum within the hippocampal complex. Taking this into consideration, the analytical Q-ball model (Descoteaux et al. 2007; Descoteaux 2010) was adopted in our study. This model relies on the decomposition of the DW signal onto a modified spherical harmonics basis, linked to the decomposition of the ODF onto the same basis by the Funk–Hecke matrix. Its use is relevant in our case, because preclinical diffusion MRI allows one to use high diffusion sensitizations ($\ge \,4500\ \hbox {s}/\hbox {mm}^2$) with short echo times (thus preventing severe $T_{2}$-decay and consequently SNR loss) yielding sharp ODFs. The DTI model was also computed to compare with the aQBI model.

#### Inference of the structural connectivity using diffusion MRI

The diffusion analyses were performed using the Connectomist toolbox. The HARDI data set was used to compute fields of ODFs stemming from the analytical Q-ball model and from the DTI model, as well as conventional quantitative maps stemming from the tensor model, including the FA map, the MD maps, and the color-encoded direction (CED) map. The analytical Q-ball model reconstruction was applied using a spherical harmonics order 8 and a regularization factor $\lambda = 0.006$, as defined in Descoteaux et al. (2007).

A streamline regularized deterministic (SRD) and a streamline regularized probabilistic (SRP) tractography algorithms (Perrin et al. 2005) were applied to the whole sample using the maps of aQBI and DTI ODFs previously computed, with the following parameters: 8 seeds per voxel yielding dense tractograms containing 11.6 millions of fibers, forward step $70\,\upmu \hbox {m}$, maximum solid angle $30^{\circ }$, minimum/maximum fiber length 0.5/100 mm to avoid loops, and discard spurious fibers. This results in four tractograms (DTI/SRD, QBI/SRD, DTI/SRP, and QBI/SRP) that will be used to analyze the differences in the inference of the structural connectivity with respect to the model and to the fiber tracking algorithm.

Following the connectomics approach introduced in Hagmann (2010) to macroscopically describe the level of connectivity between two sets of regions of interest, we computed, for the deterministic and the probabilistic tractogram obtained with the aQBI model, a $22 \times 22$ connectivity matrix that, for each pair of hippocampal subfields, counts the number of connections present in the former tractogram and linking them. The matrix is symmetric, because efferent and afferent projections cannot be distinguished with diffusion MRI, and the exclusion of self-connections implies a zero diagonal line. This connectivity matrix gives a concise overview of the main hubs of connections present in the hippocampus. In practice, whole brain studies performed on clinical systems rely on data suffering from low SNRs at high *b* value, causing overweighting of short reconstructed fibers with respect to long ones. A simple way to counterbalance such effects is to normalize each point of the matrix by the logarithm of the average length of fibers connecting to the concerned subregions. In our case, the hippocampus remains a small structure internally connected with relatively short fibers (average length 34.15 mm; standard deviation of length 21.85 mm) and tractography was relying on a high SNR diffusion MRI data set, thus less prone to fiber tracking degenerescence. Consequently, there is no need to apply any normalization of the connectivity matrix.

Finally, elements of the trisynaptic pathway, one of the most extensively studied pathways of the brain, were reconstructed from the four tractograms. The trisynaptic pathway, as presented in Duvernoy (2005), is composed of three elements:

1. Perforant path: axons from neurons in the entorhinal cortex that make synaptic contacts with dendrites of the granule cells in the dentate gyrus, located in the molecular layer of the dentate gyrus.
2. Mossy fibers: the granule cells of the dentate gyrus project to the pyramids of the CA3 (and partly also CA2) region. Synaptic contacts are located in the lucidum layer, which is between the pyramidal and radiatum layers of CA3.
3. Schaffer collaterals: pyramids of the CA3 region send their axons via the alveus–fimbria–fornix to the mammillary bodies. In addition, collaterals of these axons terminate on the dendrites of CA1 pyramids. These synaptic contacts are located in the lacunosum-molecular layer of CA1.

The four tractograms (QBI/SRP, QBI/SRD, DTI/SRP, and DTI/SRD) were used to extract mossy fibers and the perforant pathway with the aim of performing a comparison of the accuracy of aQBI versus DTI as well as SRD versus SRP tractography algorithms in reconstructing known pathways. To extract trisynaptic elements from the four tractograms, analysis pipelines were developed to intersect the connectograms with the starting and ending regions of interest corresponding to the termination of each element, plus a set of intermediate regions crossed by fibers to avoid the selection of false positives. The four tractograms were analyzed using a single filtering pipeline for each pathway.

#### Inference of tissue microstructure using diffusion MRI

Because of its practical implementation, from the acquisition point of view with respect to alternative models such as ActiveAx and AxCaliber, the NODDI model has become very popular to map the tissue microstructure in vivo in the frame of clinical applications (Kunz 2014; Chang 2015; Jelescu et al. 2015; Kodiweera et al. 2016). This model was specifically designed to map the biodistribution of dendrites and axons in the brain. It has been mostly applied in vivo in human using low-field conventional MRI scanners, limited to the millimeter spatial resolution. However, some ex vivo preclinical studies have also been performed using NODDI in animal models [mice in Sepehrband et al. (2015) and monkeys in Alexander et al. (2010)]. However, to our knowledge, this is the first time that this model is used to explore the human hippocampus ex vivo. The NODDI model consists of four diffusive compartments (Alexander et al. 2010; Zhang et al. 2012) with no exchange between them. Each compartment contributes to the global diffusion attenuation *A* resulting from a linear combination of the individual signal attenuations associated with each compartment:

1. $A_{\text {ic}}$, the signal attenuation stemming from the compartment of highly restricted water molecules trapped within axons and dendrites (i.e., neurites) modeled as cylinders of zero diameter (i.e., sticks) and characterized by a volume fraction $f_{\text {ic}}$,
2. $A_{\text {ec}}$, the signal attenuation stemming from the extra-cellular compartment of water molecules surrounding the neurites characterized by a volume fraction $f_{\text {ec}}$. This compartment is modeled by a cylindrically symmetric tensor, assuming a Gaussian anisotropic diffusion independent from the diffusion time,
3. $A_{\text {iso}}$, the signal attenuation stemming from the CSF compartment containing free molecules with an isotropic displacement probability and characterized by a volume fraction $f_{\text {iso}}$,
4. $A_{\text {stat}}$, the signal attenuation stemming from the compartment of stationary water molecules trapped within glial cells modeled as spheres of zero diameter (i.e., points) and characterized by a volume fraction $f_{\text {stat}}$. This additional compartment results from the process of fixation, in particular with the $4\%$ formaldehyde, that reduces the membrane permeability of glial cells due to an interaction with aquaporin channels (Thelwall et al. 2006).

The net diffusion signal attenuation *A* corresponds to the following linear combination:

3 $$\begin{aligned} A = f_{\text {ic}}\cdot a._{\text {ic}} + f_{\text {ec}}\cdot a._{\text {ec}} + f_{\text {iso}}\cdot a._{\text {iso}} + f_{\text {stat}}\cdot a._{\text {stat}} \end{aligned}$$

with $$f_{\text {ic}} + f_{\text {ec}} + f_{\text {stat}} + f_{\text {iso}} = 1$$.

The signal from the stationary population remains unattenuated by diffusion weighting, yielding $A_{\text {stat}} = 1$. Equation (3) can, therefore, be written as follows:

4 $$\begin{aligned} \begin{aligned} A&=(1-f_{\text {iso}}) \left[ f_{\text {ic}}^{'}\cdot a._{\text {ic}}+f_{\text {ec}}^{'}\cdot a._{\text {ec}} + f_{\text {stat}}^{'} \right] + f_{\text {iso}}\cdot a._{\text {iso}}\\&=(1-f_{\text {iso}})\left[ (1-f_{\text {stat}}^{'})(f_{\text {ic}}^{*}\cdot a._{\text {ic}}+f_{\text {ec}}^{*}\cdot a._{\text {ec}}) + f_{\text {stat}}^{'} \right] \\&\quad +\,f_{\text {iso}}\cdot a._{\text {iso}}\\ \end{aligned} \end{aligned}$$

with $f_{\text {ic}}^{'} + f_{\text {ec}}^{'} + f_{\text {stat}}^{'} = 1$ and $f_{\text {ic}}^{*} + f_{\text {ec}}^{*} = 1$, hence:

5 $$\begin{aligned} \begin{aligned} A&= (1-f_{\text {iso}})\left[ (1-f_{\text {stat}}^{'})(f_{\text {ic}}^{*}\cdot a._{\text {ic}}+(1-f_{\text {ic}}^{*})A_{\text {ec}}) + f_{\text {stat}}^{'} \right] \\&\quad +f_{\text {iso}}\cdot a._{\text {iso}}\\ \end{aligned} \end{aligned}$$

with $$f_{\text {stat}} = (1-f_{\text {iso}}) f_{\text {stat}}^{'}$$ and $$f_{\text {ic}} = (1-f_{\text {iso}})(1-f_{\text {stat}}^{'}) f_{\text {ic}}^{*}$$.

To speed up the fitting procedure, some parameters were fixed as suggested by Zhang et al. (2012). Watson’s distribution was preferred to Bingham’s distribution. Whereas, for in vivo studies, intrinsic and isotropic diffusivities are usually set to 1.7 $\times 10^{-3}$ and $3.0 \times 10^{-3}\ \hbox {mm}^{2}/\hbox {s}$, respectively (Zhang et al. 2012), they were set to $0.16 \times 10^{-3}\ \hbox {mm}^{2}/\hbox {s}$ corresponding to the mean diffusivity found in the grey matter of our sample and to the diffusion coefficient of water at $20\,^{\circ }\hbox {C}$, i.e., $2.0 \times 10^{-3} \hbox {mm}^{2}/\hbox {s}$.

Statistics of the intra-cellular volume fraction $f_{\text {ic}}$ (assumed to represent the neurite density) were analyzed for each segmented structure of the hippocampus from the histogram of its values within the structure, to study their variation according to the structure of interest.

## Results

### Anatomical MRI/3D rendering of anatomical hippocampal structures

**Fig. 2:**
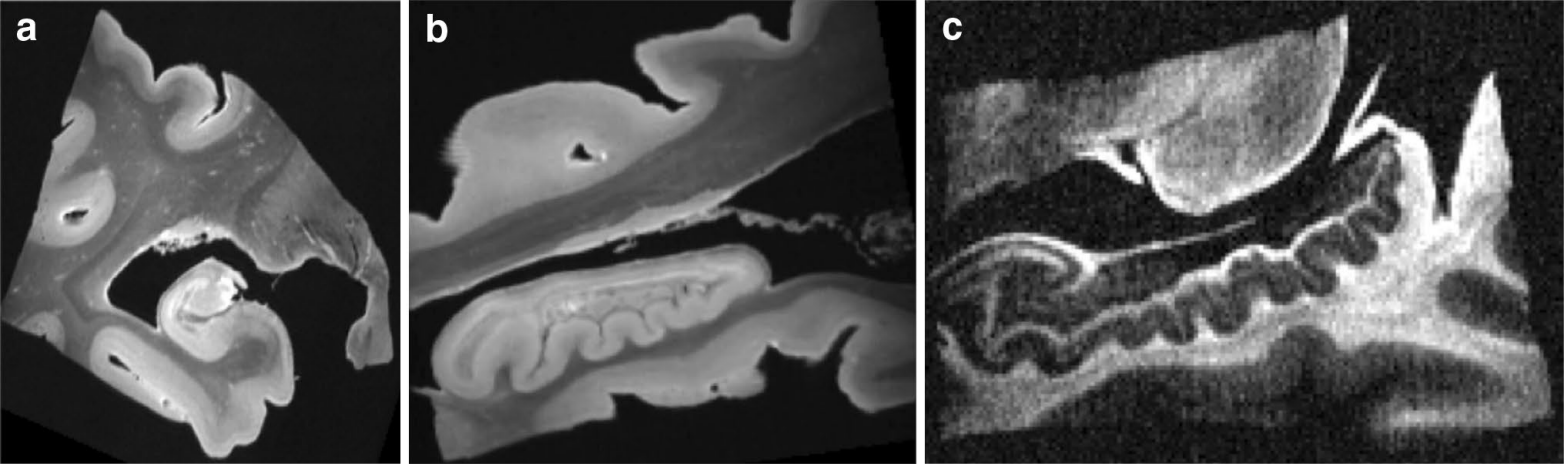
Raw images obtained with the anatomical acquisition at $200\ \upmu \hbox {m}$ (**a**, **b**), and with the diffusion acquisition at ${b} = 4500\ \hbox {s}/\hbox {mm}^{2}$ (**c**)

**Fig. 3:**
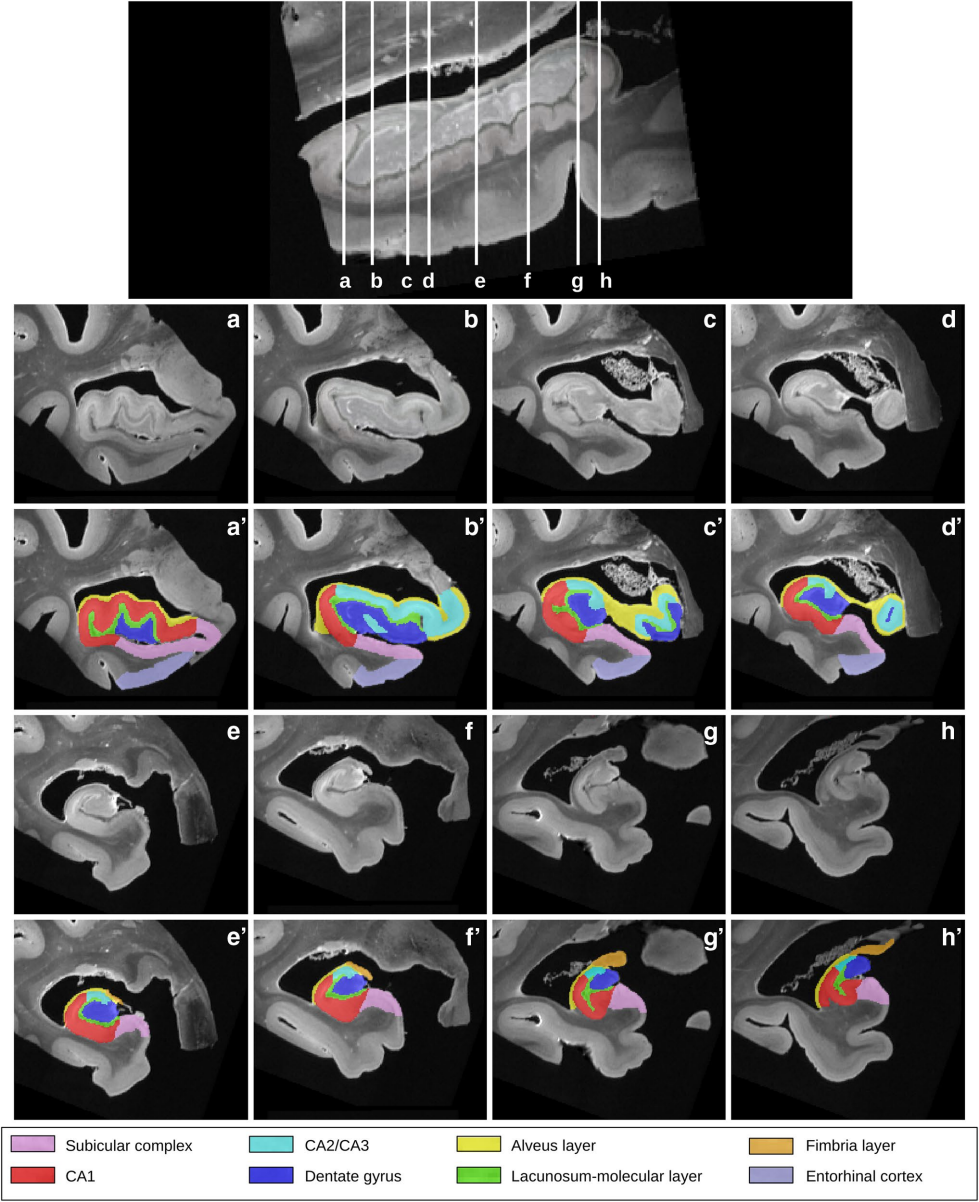
The upper figure shows a sagittal view with references to all the coronal images. Coronal images of the hippocampal formation are shown in an anterior-to-posterior direction from a to h. The head is displayed in **a**–**d**, the body in **e** and **f**, and the tail in **g** and **h**. The segmentation is shown in **a**$^{'}$–**h**$^{'}$

The anatomical T2-weighted MRI data set ($200\ \upmu \hbox {m}$, Fig. 2a, b) presented a very good contrast and SNR (33.7), thus enabling accurate delineation of the entorhinal cortex and of several components of the hippocampal complex: dentate gyrus, pyramidal and radiatum layers of the CA1 and CA2/CA3 regions, lacunosum-molecular layer, alveus, fimbria, and subicular complex. Figure 3 shows series of coronal sections with all the delineated areas. A three-dimensional rendering of the manual segmentation of the hippocampal regions and layers as well as of the entorhinal cortex is available in Supplementary Material. The accuracy of the segmentations is a key factor to successfully discriminate the fiber tracts connecting them.

### DTI and Q-ball imaging

**Fig. 4:**
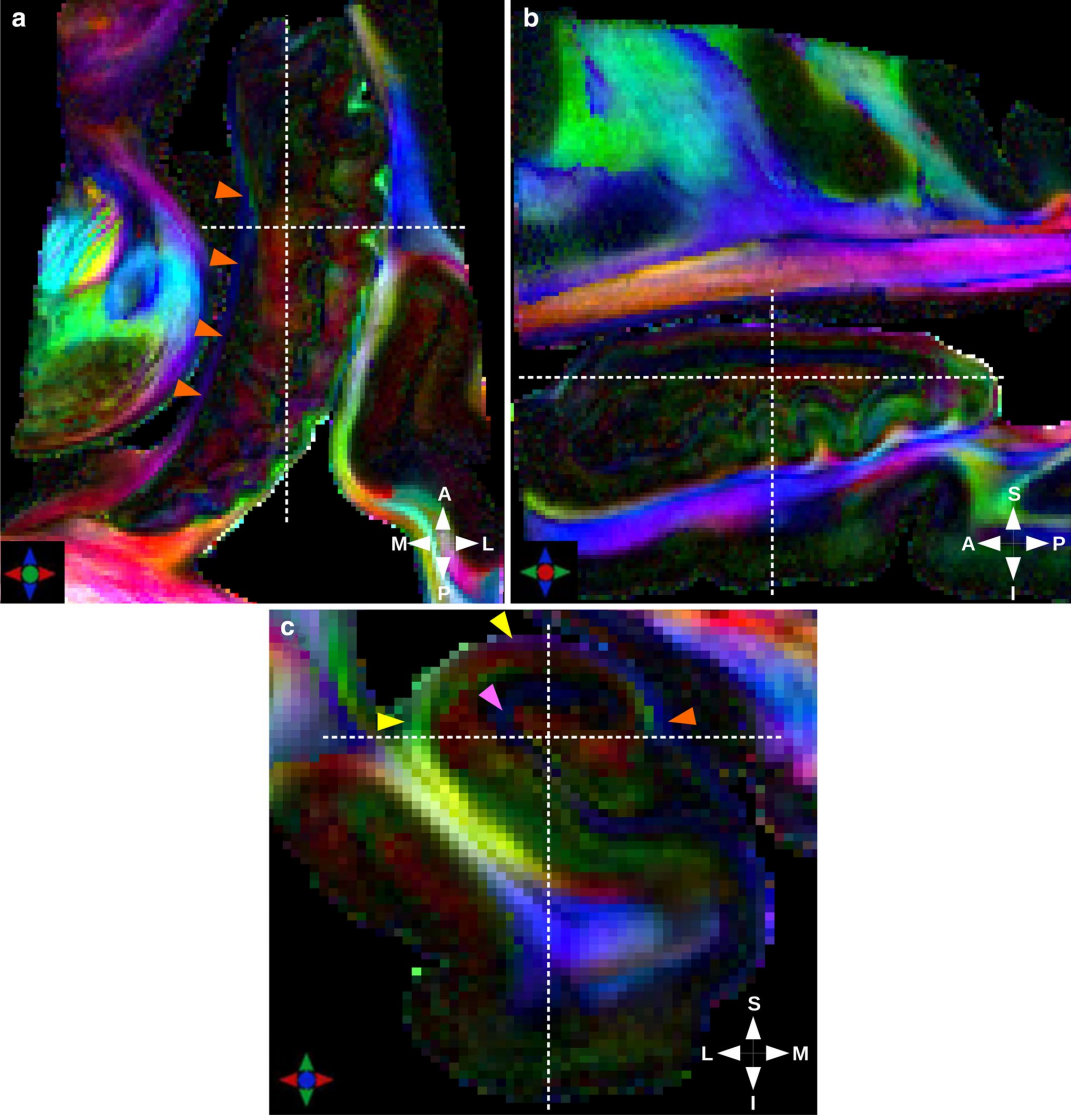
Color-encoded diffusion directions at $300\,\upmu \hbox {m}$ and 500 directions. The white dotted lines indicate the correspondence between axial (**a**), sagittal (**b**), and coronal (**c**) slices. Orange arrowheads point at the fimbria, yellow arrowheads at the alveus, and pink arrowhead at the lacunosum-molecular layer. The color-encoding cross at the bottom left of the image depicts the direction of largest displacement probability orientation. ${A} \, \hbox {anterior}$, ${P}\, \hbox {posterior}$, ${M}\, \hbox {medial}$, ${L} \,\hbox {lateral}$, ${S}\, \hbox {superior}$, ${I} \,\hbox {inferior}$

**Fig. 5:**
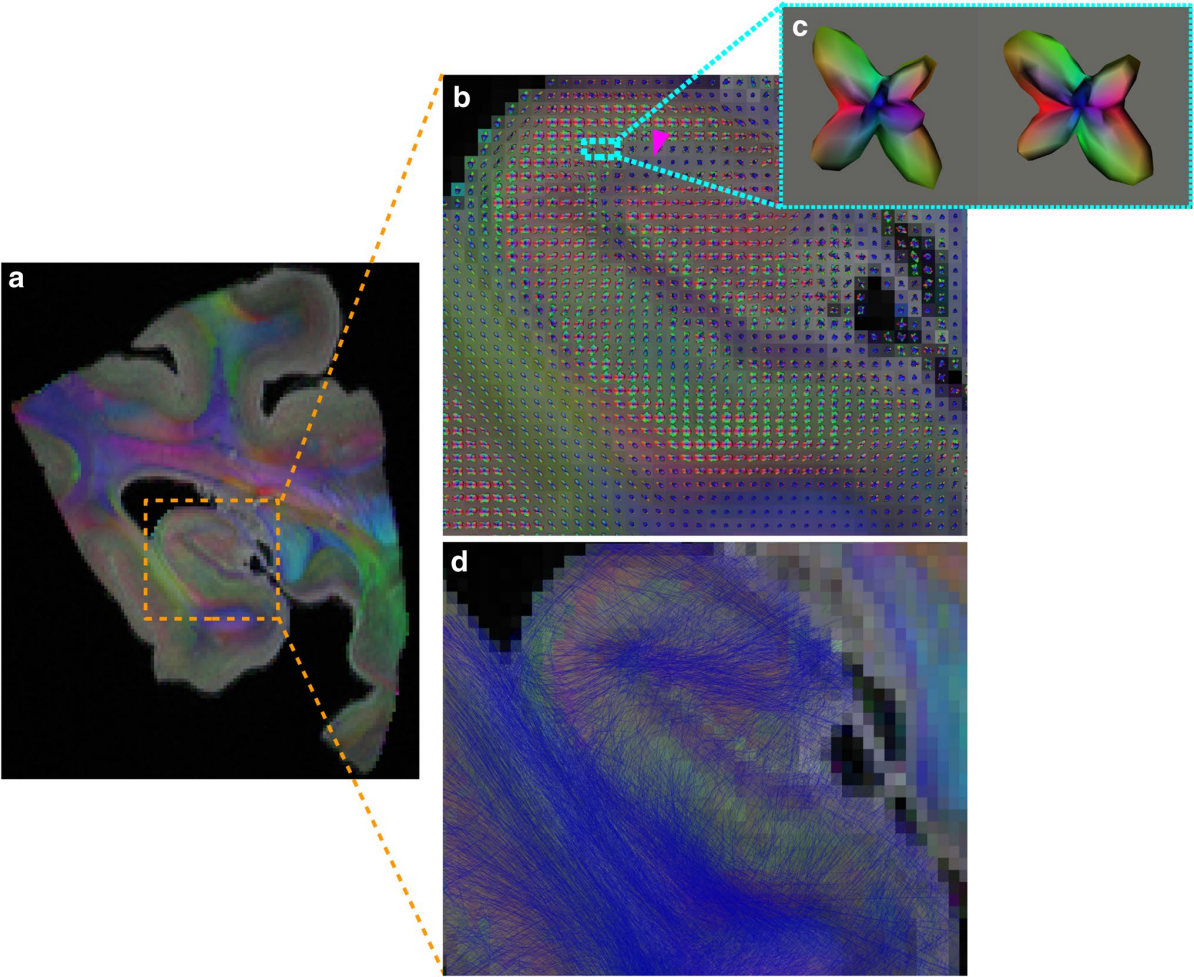
On the left, fusion of the $T_{2}$(${b}=0$) image and the color-encoded diffusion directions map obtained with the DTI model (**a**). The color-encoding cross at the bottom left depicts the direction of largest displacement probability orientation. On the right, the orientation diffusion functions field computed with the Q-ball model (**b**) with a zoom of ODFs showing a crossing at the level of the Schaffer collateral (**c**), and the SRP tractogram calculated with this field (**d**), superimposed on (**a**). Pink arrowhead point at the radiatum and lacunosum-molecular layers with ODF orthogonal to the coronal plane

Figures 4, 5 depict the obtained color-encoded direction map, as well as the Q-ball ODF field and the tractogram obtained with a probabilistic algorithm superimposed on the $T_{2}$(${b}=0$) reference map. The two figures were obtained using the HARDI data set. The color-encoded maps shown in Fig. 4 reveal a plethora of fine anatomical details and the ODF peaks shown in Fig. 5b, c seem in good agreement with the underlying structural connectivity.

The high anisotropy in the fimbria, oriented in the sagittal plane (orange arrowheads in Figs. 1, 4), can be related to efferent axons from CA3, CA1, and the subicular complex, along with afferent axons from structures in the diencephalon and basal forebrain. These fibers run parallel to the septal–temporal axis of the hippocampus. This main orientation is also visible in Fig. 4a, where the fimbria appears in blue near the head, then pink, and almost red when it goes towards the fornix.

Regarding the alveus, oriented in the coronal plane (yellow arrowheads in Figs. 1 and 4), it contains the axons from the CA1 region and the subicular complex, reaching the fimbria through the alveus in an oblique septal direction. The warping of the fibers around the surface of the hippocampus is also visible in Fig. 4c, where the alveus appears green and then pink when it gets close to the fimbria.

The fiber orientation observed in the pyramidal layer (Figs. 4, 5) can be attributed to the projection of the large apical dendrites of CA1 and CA3 through the lucidum (only in CA3) and radiatum layers towards their termination in the lacunosum-molecular layer. It is also affected by the perforant pathway. Orthogonally to the projection of CA1, CA2, and CA3 dendrites towards the lacunosum-molecular layer, the Schaffer collaterals runs from CA3 to CA1 (probably corresponding to the red part in the pyramidal layer from CA3 to CA1 in Fig. 4c). Voxels between CA3 and CA1 contain multiple fiber crossings, which cannot be resolved by the DTI model. This demonstrates the relevance of using the HARDI/Q-ball model. Figure 5b shows ODFs recovering multiple fiber crossings in the pyramidal layer with a shape revealing two main peaks (one for each principal direction). Figure 5c clearly depicts ODFs with two main peaks that can also be attributed to the Schaffer collateral crossing the projection of CA1, CA2, and CA3 dendrites towards the lacunosum-molecular layer.

In the lacunosum-molecular layer, and to a lesser extent in the radiatum layer, the apical dendrites of CA-pyramids diverge orthogonally into the terminal arborizations that tend to run parallel to the hippocampal sulcus. This corresponds to the area in the radiatum and lacunosum-molecular layers with ODF orthogonal to the coronal plane (pink arrowheads in Figs. 4c, 5b).

Hence, although color-encoded maps obtained with the DTI model show the main directions in each voxel and lead to a partial understanding of the fibers pathways, HARDI/QBall model is mandatory to resolve fiber crossings involved in the Schaffer collaterals and the perforant pathway.

### Analysis of the connectivity matrix and reconstruction of elements of the polysynaptic pathway

**Fig. 6:**
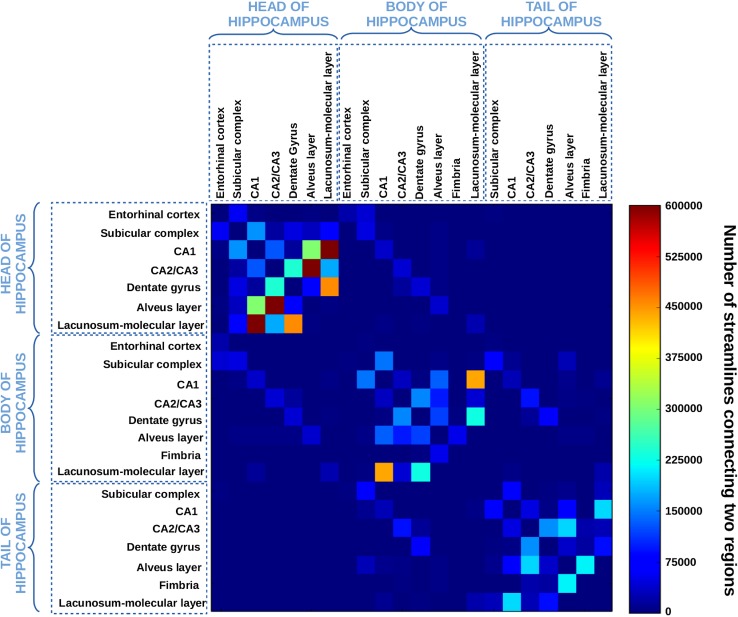
Connectivity matrix of the hippocampal substructures obtained with the streamline regularized deterministic model. Each matrix element represents the number of fibers connecting the ROIs indicated by the column and by the row. Self-connections are excluded, which implies a zero diagonal line in the matrix. The map is symmetric, because efferent and afferent projections cannot be distinguished with diffusion MRI. The heat scale represents the number of fibers connecting the ROIs

**Fig. 7:**
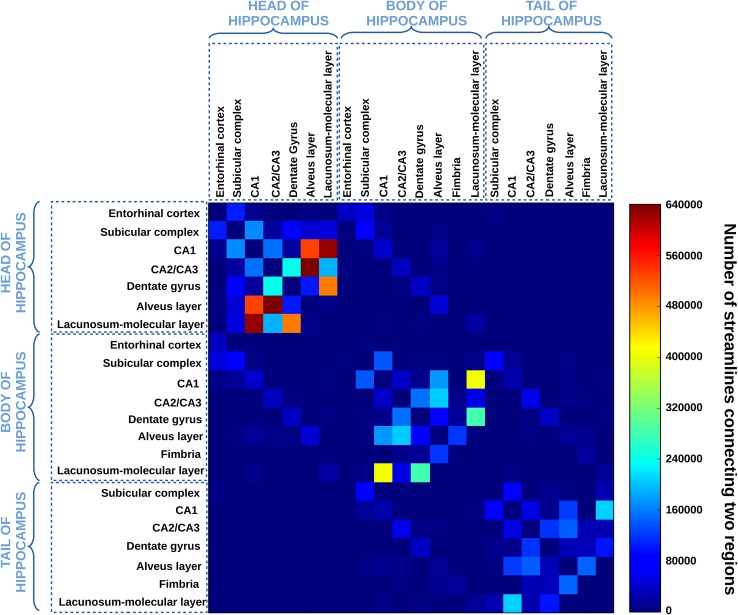
Connectivity matrix of the hippocampal substructures obtained with the probabilistic model. Each matrix element represents the number of fibers connecting the ROIs indicated by the column and by the row. Self-connections are excluded, which implies a zero diagonal line in the matrix. The map is symmetric, because efferent and afferent projections cannot be distinguished with diffusion MRI. The heat scale represents the number of fibers connecting the ROIs

Figures 6, 7 show the connectivity matrices of the hippocampal substructures, obtained with the streamline regularized deterministic (Fig. 6) and the streamline regularized probabilistic algorithms (Fig. 7) applied with Q-ball model. As a general trend, the two matrices reveal a higher level of connectivity in the head compared to the body and the tail. The high connectivity of the lacunosum-molecular layer with CA1 can be explained by the location of the apical dendrites of pyramidal neurons of CA1 in this layer. As regards the connectivity between CA1 and the alveus in the head, this is likely due to pyramids of the CA1 region that send their axons via the alveus–fimbria–fornix to the mammillary bodies and representing one source of output from the hippocampus. The same applies to connectivity between CA2/CA3 and the alveus. Moreover, the high connectivity between CA1 and the alveus can be due not only to the presence in this structure of efferent axons from CA1 pyramids, but also to the basal dendrites of the pyramidal neurons bending into the alveus, as described in both polysynaptic and direct pathways (Duvernoy 2005). It also reveals connections that extend through the length of the hippocampus. Connections within each structure are also found. The subicular complex of the body is connected with the subicular complex of the head and the subicular complex of the tail. This is also the case for the dentate gyrus or CA2/CA3. This is in agreement with a primate study (Kondo et al. 2008), showing that the projections of the dentate gyrus extend bidirectionally along much of the length of the hippocampus. Finally, longitudinal connectivity also occurs between related regions like the entorhinal cortex in the hippocampal head and subicular complex in its body. Thus, while the subfields are usually studied in the coronal plane, it appears that connections between subfields extend both in cross section and longitudinally. All these results are in agreement with a recent literature review (Strange et al. 2014) showing a gradient of connectivity that varies along the length of the hippocampus. Finally, the two matrices also depict different connectivity levels. For instance, the level of connectivity between the dentate gyrus and CA2/CA3 appears lower with probabilistic tractography than with deterministic tractography. Conversely, the level of connectivity between CA1 and the alveus is lower with the deterministic approach. These observations would benefit from further analysis and comparison with a gold standard, which is beyond the scope of this study.

**Fig. 8:**
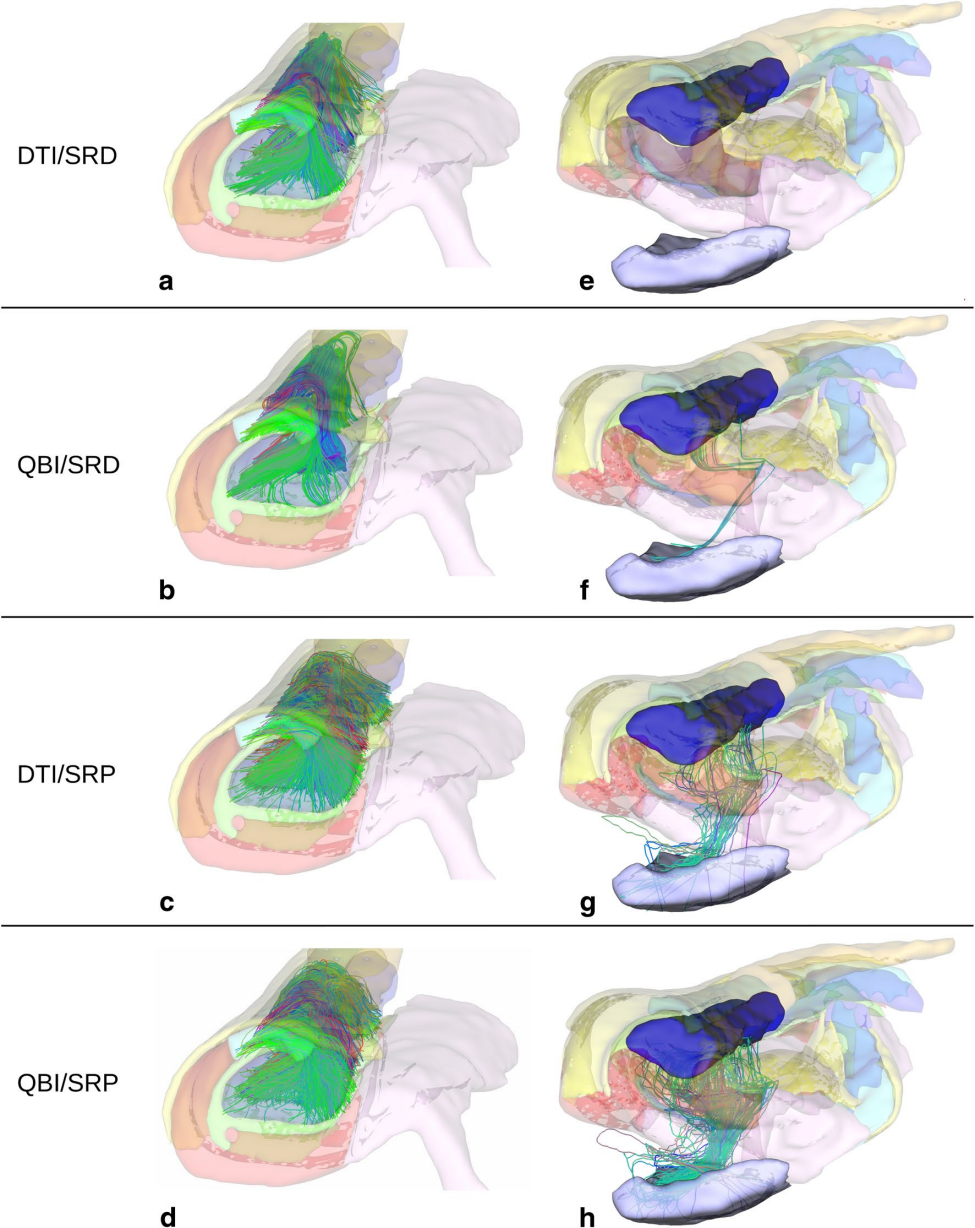
3D-rendering of the perforant pathway and mossy fibers in the body of the hippocampus extracted from four tractograms (DTI/SRD, QBI/SRD, DTI/SRP, QBI/SRP) **a**–**d** mossy fibers in the body, from the granular layer of the dentate gyrus to the lucidum layer of CA3. 3D-rendering only shows the body segmentation; **e**, **f** perforant pathway from the entorhinal cortex to the molecular layer of the dentate gyrus in the body of the hippocampus. 3D-rendering shows the entire segmentation (head, body, and tail) with the same color code as the one presented in Fig. 3 and a transparency of 0.2, except for the entorhinal cortex (opaque grey) and the dentate gyrus in the body (opaque dark blue)

Figure 8 shows how elements of the polysynaptic pathway could be extracted using high field/strong gradients diffusion MR-based tractography with the QBI/SRP tractogram, and also illustrates the differences in the inference of the structural connectivity with respect to the model and to the fiber tracking algorithm. Mossy fibers could be extracted using the four approaches. Although probabilistic approaches display fibers with a more realistic distribution of their origins along the granular layer of the dentate gyrus. By contrast, when it comes to the extraction of the perforant pathway, probabilistic approaches were the only one to give satisfying results, thus playing in favour of the use of a probabilistic fiber tracking technique. The QBI/SRD method leads to a bundle with very few fibers and the DTI/SRD tractogram does not allow the extraction of any fiber of the perforant pathway. This can be attributed to the fact that probabilistic tractography with multiple fiber orientations shows more robustness to noise and more sensitivity than the standard deterministic tractography as it explores all possible options, allowing to temporarily select suboptimal directions during the streamlining process (Behrens et al. 2007). The comparison of the QBI/SRP and the DTI/SRP results shows the benefit arising from the use of Q-ball model, since the resulting bundle displays more fibers and with a more regular distribution.

### Analysis of the hippocampal tissue microstructure

#### Investigating neurite density in the hippocampus

**Fig. 9:**
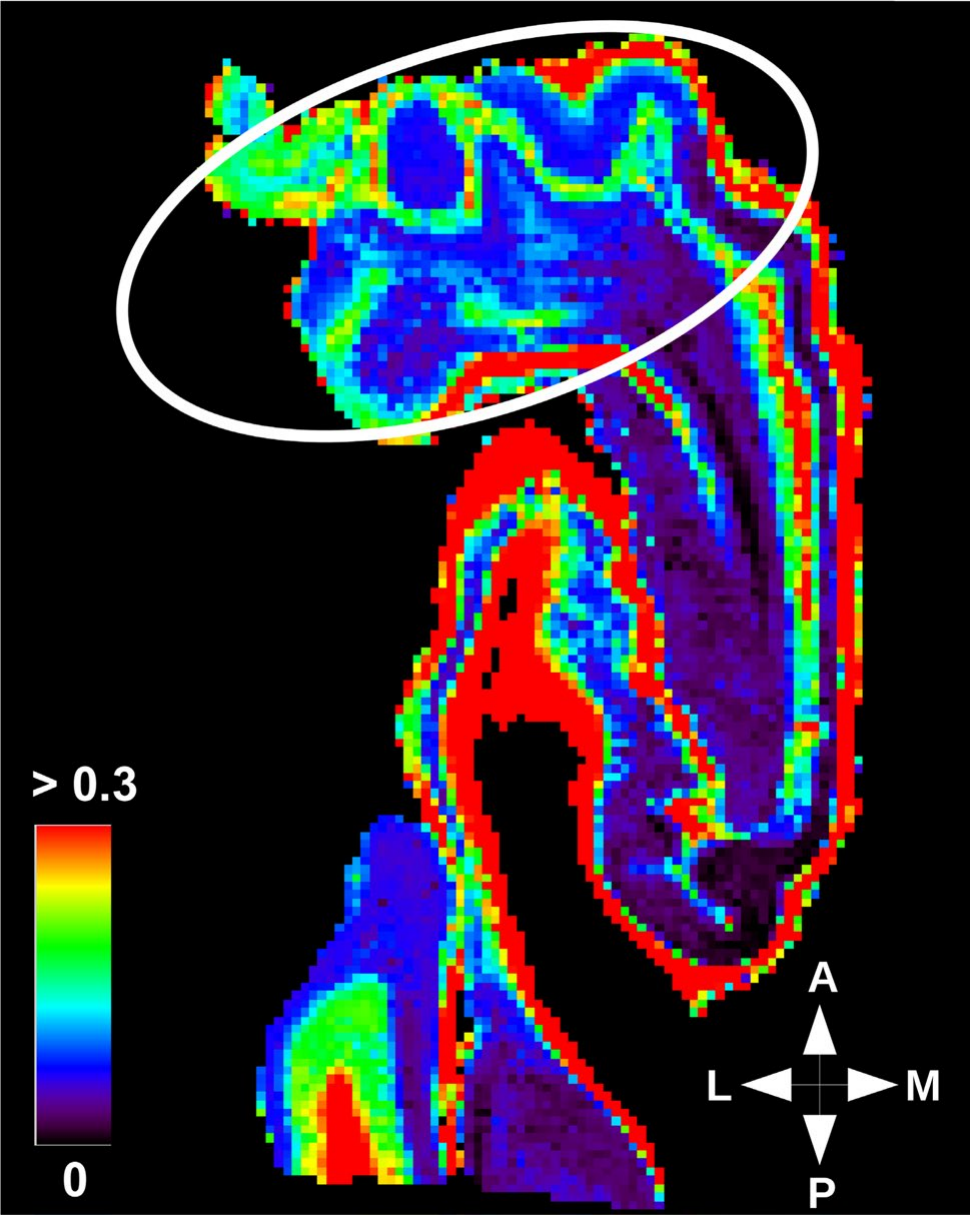
Neurite density gradient in hippocampal grey matter. The intra-cellular volume fraction obtained with NODDI model was set with a limit at 0.2 to highlight gradients in grey matter regions. White circle indicates a region of higher intra-cellular volume fraction in grey matter. The heat scale represents the intra-cellular volume fraction. ${A} \, \hbox {anterior}$, ${P}\, \hbox {posterior}$, ${M}\, \hbox {medial}$, ${L} \,\hbox {lateral}$

**Fig. 10:**
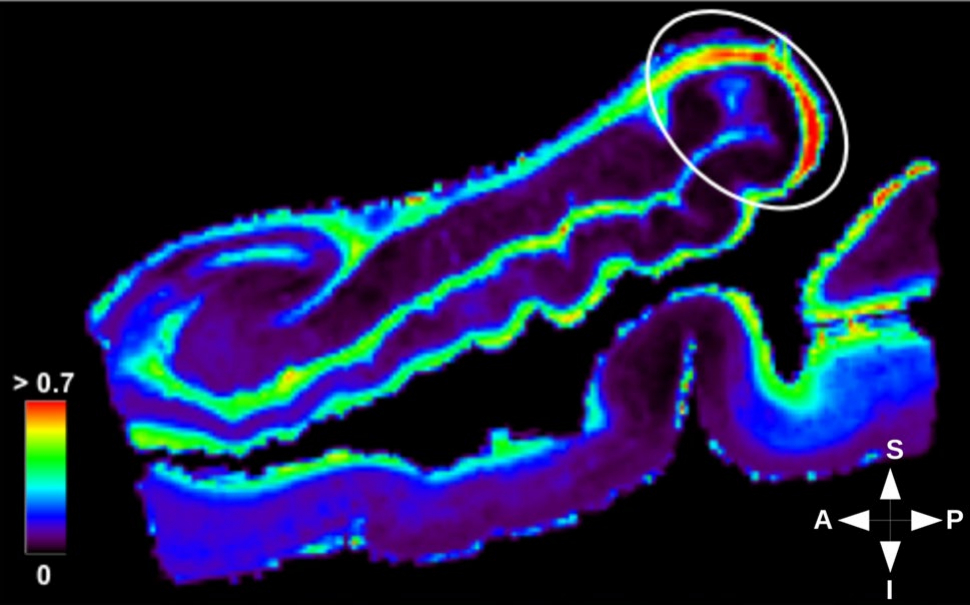
Neurite density gradient in hippocampal white matter. The intra-cellular volume fraction map obtained with NODDI model was determined with maximum limit set at 0.7 to highlight gradients in white matter regions where the axonal density is high. White circle indicates a zone of higher density in the posterior hippocampus. The heat scale represents the intra-cellular volume fraction. ${A} \,\hbox {anterior}$, ${P}\, \hbox {posterior}$, ${S} \,\hbox {superior}$, ${I} \, \hbox {inferior}$

As already mentioned, the restricted intra-cellular volume fraction inferred from the NODDI model corresponds to the neurite density. Within the hippocampus, Figs. 9 and 10 clearly depict a positive gradient of this neurite density in the grey matter from the posterior to the anterior part of the hippocampus and, conversely, a negative gradient in the white matter.

Mean intra-cellular volume fractions were computed for each segmented structure and reported in Table 2. Table 2 also assesses the positive gradient of intra-cellular fraction in the anterior part of the hippocampus, with a higher neurite density in grey matter regions, especially in the subicular complex and CA1. This result is consistent with the results obtained with the tractography, which also revealed a higher connectivity in the head than in the body or tail portions of the hippocampus (Figs. 6, 7). Only CA2/CA3 does not follow the gradient described for the grey matter regions. That might be due to the fact that cell packing density within CA2/CA3 varies along the rostro-caudal axis of the hippocampus. It has already been described to be higher in the body than in the head part in monkeys (Willard et al. 2013). Partial volume effects occurring with the segmentation can also have an impact, e.g., voxels labeled as CA2/CA3, but actually containing white matter.

Table 2 also shows the increased intra-cellular fraction in the tail, with a higher neurite density in white matter, i.e., the alveus and fimbria. This result can be interpreted on the basis of the polysynaptic pathway, since the principal outputs to the cortex merge along the different rostro-caudal levels of the hippocampus in the fimbria, which could explain the gradual increase in axonal density in the fimbria along the anterior–posterior direction.

**Table 2:** Mean intra-cellular fraction for each label

Region	Label	Mean $f_{\text {ic}}$
Head	Entorhinal cortex	0.135
	Subicular complex	0.202
	CA1	0.071
	CA2/CA3	0.082
	Dentate gyrus	0.068
	Alveus	0.196
	Lacunosum-molecular layer	0.112
Body	Entorhinal cortex	0.131
	Subicular complex	0.098
	CA1	0.030
	CA2/CA3	0.084
	Dentate gyrus	0.037
	Alveus	0.443
	Fimbria	0.269
	Lacunosum-molecular layer	0.110
Tail	Subicular complex	0.056
	CA1	0.021
	CA2/CA3	0.099
	Dentate gyrus	0.036
	Alveus	0.479
	Fimbria	0.604
	Lacunosum-molecular layer	0.074

#### Investigating the laminar structure of the Ammon’s horn

**Fig. 11:**
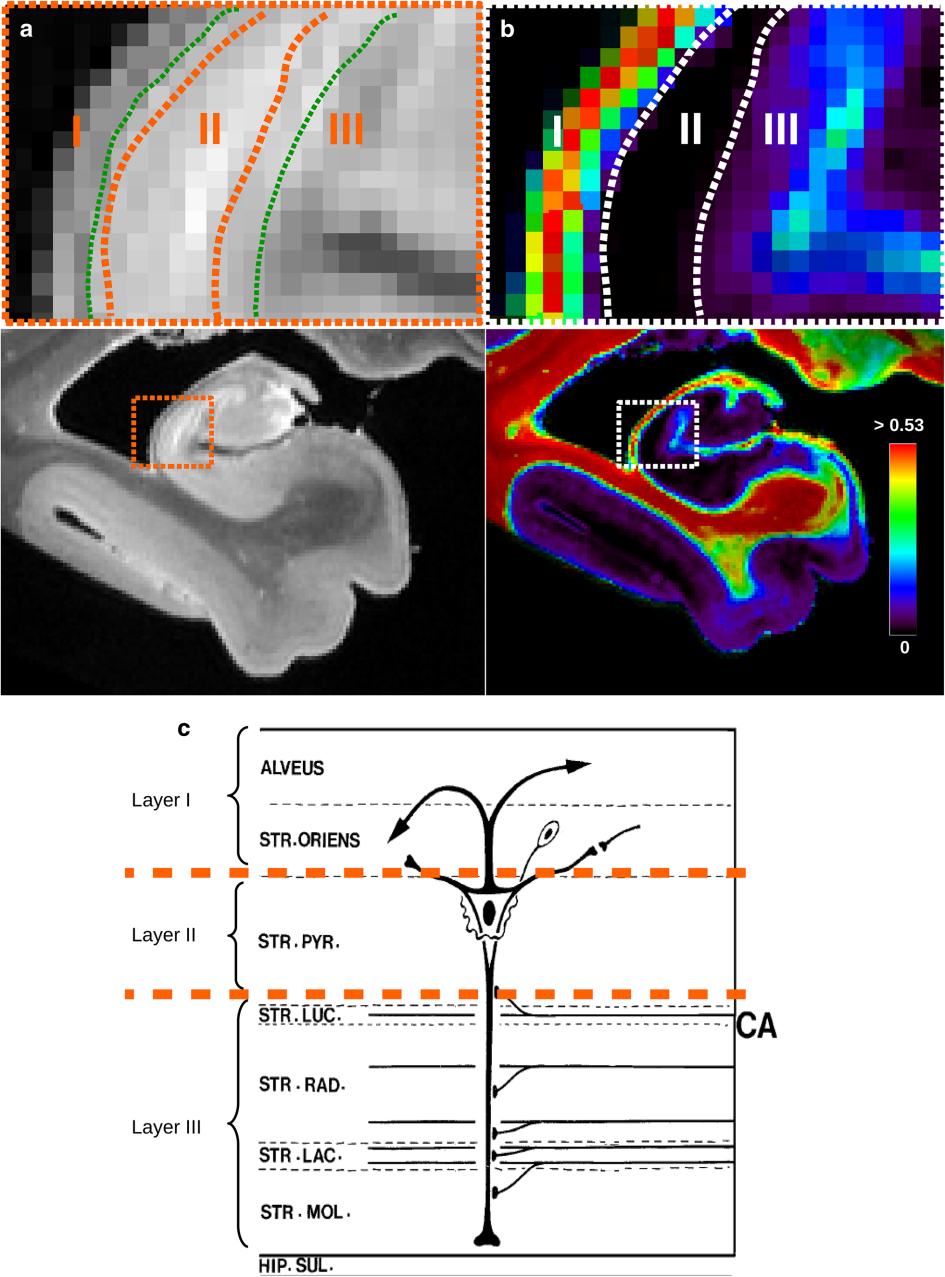
Comparison of the contrast between the three hippocampal layers using **a** a standard anatomical $T_{2}$-weighted spin-echo sequence and **b** the intra-cellular volume fraction map obtained with NODDI model. The heat scale represents the intra-cellular volume fraction. The theoretical layers in cornu Ammonis are drawn in **c**, adapted from Duvernoy (2005). Their boundaries have been recognized, based on the T2-weighted image, with the green dotted lines in **a** and also with the help of the intra-cellular volume fraction map as shown with the orange dotted lines in **a** and the white dotted lines in **b**

The main hippocampal layers, referred to as layer I, II, and III in Fig. 11c taken from Duvernoy (2005), can be segmented from $T_{2}$-weighted images, but their contrast does not provide an indisputable boundary for each layer. As depicted in Fig.  11a, the boundary remains partially defined. Green dotted lines are the boundaries inferred only with the $T_{2}$-weighted contrast and, in orange, limits obtained with the combination of the T2-weighted image and the neurite density map (Fig. 11b). Figure 11b clearly demonstrates that the intra-cellular volume fraction of the neurite population significantly enhances the contrast between layers and facilitates their segregation. This is particularly true when considering the delineation of the boundary separating layers II and III. Furthermore, the underlying physical principle driving this novel contrast mechanism is coherent with the anatomical knowledge. Layer I, with a high neurite density, corresponds to the alveus and to the oriens layers. Layer II, in contrast, shows a very poor neurite density, and corresponds to the pyramidal layer, mostly composed of the somas of neurons. Layer III, adjoining the vestigial hippocampal sulcus, appears with a higher neurite density than the pyramidal layer, which is consistent with the fact that it contains arborizations of the apical dendrites of pyramidal neurons, and corresponding to the molecular zone of the CA region, i.e., to the radiatum and lacunosum-molecular layers.

The intra-cellular volume fraction map thus shows new contrasts, consistent with histology compartments, that could be applied in the future to other cortical brain to improve the quality of the segmentation at the level of cortical layers.

## Discussion

The main contributions of this work are:

1. The presentation of a novel ultra-high field $T_{2}$-weighted and diffusion MRI protocol providing high spatial and angular resolution data. It appears to be useful to propose a novel segmentation approach of the human hippocampus subfields that combines the available sources of contrast to enhance their segregation.
2. A proof-of-concept that ultra-high field high angular resolution diffusion imaging (UHF-HARDI) can robustly map the inner connectivity of the hippocampus and give evidence of a higher level of connectivity in the uncal region than in the body or tail portions. This result holds regardless of the tractography approach. The inference of known pathways was provided to illustrate the potential of UHF-HARDI. The comparison between the results obtained from the four tractograms highlights the need for using a probabilistic tractography algorithm and, when it comes to the reconstruction of more complex pathways like the perforant pathway, the benefit arising from the use of Q-ball model.
3. A proof-of-concept that ultra-high field diffusion MR microscopy could play an increasing role in the future to decipher the cytoarchitecture of the hippocampus. In our case, it provides evidence of a rostro-caudal heterogeneity that could be associated with differences in gene expression patterns and could support the long-axis functional specialization of the human hippocampus (Strange et al. 2014). Indeed, the anterior portion of the hippocampus, but not its posterior one, is activated by tasks probing emotional and motivational aspects of cognitive processes (Kim and Fanselow 1992; Viard et al. 2011). Furthermore, whereas the caudal portion of the hippocampus is involved in the encoding of memories or in the local component of spatial representation, its rostral part plays a crucial role in retrieval processes and in the global component of spatial representation (Poppenk et al. 2013; Zeidman et al. 2015; Zeidman and Maguire 2016).

### Comparison with existing studies

Several studies have been published to design a robust segmentation pipeline of the hippocampal substructures. Most of those relying on MRI were designed to exploit a single contrast, either $T_{2}$ or $T_{2}^{*}$ weighting (Wisse et al. 2012; Boutet 2014; Adler et al. 2014; Yushkevich 2009). The segmentation pipeline proposed in this study combines several contrasts stemming from $T_{2}$-weighted anatomical MRI and diffusion-weighted MRI to take benefit from all the available information. Not only the scalar information of diffusion MRI data were exploited, but also the angular profiles of ODFs. Their singularities could be used to better identify the boundaries between substructures of the hippocampal formation when the $T_{2}$ contrast did not provide enough valuable information, e.g., the transition from alveus to fimbria.

Most existing studies aiming at investigating hippocampal microstructure rely on a diffusion imaging protocol acquired using a single-shell sampling of the *q*-space, therefore, not compatible with the use of the novel models emerging from the field of diffusion MR microscopy, requiring multiple-shell HYDI acquisition schemes. Most of them used the diffusion tensor model (DTI) and investigated the variations of FA, ADC, or just the contrast of the $T_{2}$-weighted image between subregions and/or the layers of the hippocampus (Shepherd et al. 2007; Coras 2014). Unfortunately, those invariant DTI-based scalar features are not specific to a particular cellular organization. A decrease in FA may correspond either to the degenerescence of axons, or to their spreading. A decrease of the ADC may correspond either to a reduction of the axon diameter or to disorders in the extra-cellular space. For instance, Coras (2014) showed the seven hippocampal layers (i.e., alveus, sr pyramidale, sr radiatum, sr lacunosum, sr moleculare, as well as the sr moleculare and granule cell layer of the DG) identified with the contrast of an $T_{2}$-weighted image for a healthy sample. Sclerotic hippocampal samples depicted only four layers, and the non-specificity of the method prevented from establishing the causes of this alteration. The HYDI acquisition scheme implemented in our study allowed us to use more advanced models of diffusion MRI giving more specific features to characterize the microstructure, like the neurite density inferred from the NODDI model. Such quantitative features lead to a better understanding of the variations occurring in the rostro-caudal direction at the cellular level and make correlations with the long-axis functional specialization of the human hippocampus.

Established pathways have already been reconstructed or identified in other studies. Zeineh et al. (2012) reconstructed, from in vivo data, the best-known pathways of the medial temporal lobe including the perforant pathway and the Schaffer collaterals. Coras (2014) also identified the perforant pathway on a DTI-based tractogram and Augustinack (2010) reconstructed it from ex vivo DTI-based data. However, our study was one of the first to go beyond diffusion tensor imaging to probe the circuits and the inner connectivity of the human hippocampus. Assuming a Gaussian distribution of water molecule displacements, DTI can support only one fiber population per voxel, thus being unable to render complex fiber configurations like crossings, kissings, or splittings. HARDI models, to which the analytical Q-ball belongs, were designed to go beyond this limitation, and are particularly suitable for the hippocampus where such configurations are likely to happen. Finally, it is the first time that connectivity matrices were used to assess the gradient of connectivity existing along the rostro-caudal axis of the hippocampus. Such observations were hypothesized from functional studies, but to our knowledge, have never been investigated from a structural point of view using diffusion MRI and tools coming from the field of connectomics.

### Limitations

In this study, we have established methods to delineate subfields and substructures of the hippocampal formation to infer their structural connectivity and their microstructure. We have given evidence that using diffusion-based microstructural maps enables the segmentation of smaller hippocampal structures, like its lamination. Investigating this potential should be generalized in the future to develop a novel MRI-based post-mortem atlas of the hippocampal complex. We have also demonstrated that UHF diffusion MRI using a preclinical system allows the reconstruction of hippocampal known pathways, like the perforant path and the mossy fibers. To go a step further, clustering techniques applied to the connectogram would provide clusters of co-localized fibers sharing similar geometries and belonging to the same white matter tract. Combined with the integration of more samples to better capture the intersubject variability, fiber clustering should accelerate the construction of a probabilistic atlas of the hippocampal inner connectivity. However, this construction is out of the scope of this paper.

In the present study, the sample is fixed by immersion, and there is a risk for the fixation to be inhomogeneous, as the fixative has to diffuse from the surface to the deepest structures. The time it takes for fixative to permeate through the brain can lead to higher fixation time for the deepest structures, inducing higher degradation of the tissue, for instance from autolysis. This inhomogeneity could be a confound when reporting gradients in microstructure maps. To minimise this risk, the sample was immersed 2 years, which is enough to entirely fix the tissue. In case of high inhomogeneities resulting from the fixation, clear non-anatomical borders would appear in the structural MRI images. Since no kinds of severe contrasts (independent from anatomical structures) were observed in the high-resolution MRI measurements, the homogeneity of the fixation can be assumed. In addition, the hippocampus is located in the periphery of the brain. There is then little risk of fixation inhomogeneities. However, it is impossible to completely eliminate this confound with one specimen and a further study would benefit from having more samples.

Another aspect that could impact the diffusion contrast and the quality of our results is the choice of the diffusion sensitization. Given the reduction factor of the mean diffusivity from ${D} = 0.7\times 10^{-3}\ \hbox {mm}^2/\hbox {s}$ (standard value reported in vivo) to ${D} = 0.16\times 10^{-3}\ \hbox {mm}^{2}/\hbox {s}$, the diffusion attenuation at ${b}=4500\,\hbox {s}/\hbox {mm}^{2}$ should be equivalent to that of an in vivo scan performed at ${b}=1000\ \hbox {s}/\hbox {mm}^{2}$. A higher diffusion sensitization is generally recommended to obtain sharp ODFs (Hess et al. 2006). The use of a single shell at $4500\,\hbox {s}/\hbox {mm}^2$ was motivated by the literature, that typically mention the use of *b* values of at least $4000\,\hbox {s}/\hbox {mm}^2$ to scan post-mortem pieces. In particular, Dyrby et al. (2011) demonstrated that any HARDI acquisition with a *b* value between 2000 and $8000\,\hbox {s}/\hbox {mm}^2$ allows for the inference of multiple fiber populations from ex vivo fixed specimens scanned at room temperature, with an optimal value around $4000\,\hbox {s}/\hbox {mm}^2$. Furthermore, the application of a probabilistic fiber tracking method contributed to more robustly manage fiber crossings than deterministic approaches. It would also be of great value to investigate alternative reconstructions using the acquired HYDI data set, to go beyond HARDI models. Advanced models could be considered, like the mean average propagator (MAP-MRI) reconstruction (Özarslan et al. 2013) or the fiber orientation distribution (FOD) reconstruction (Jeurissen et al. 2014), relying both on a multiple-shell sampling of the *q*-space. On the one hand, MAP-MRI would provide the estimation of further information like the return-to-origin probability, sensitive to compartment sizes, or non-Gaussianity, providing insights about the tissue complexity. On the other hand, FODs inferred from multiple-shell acquisitions are based on a multi-tissue constrained spherical deconvolution that would provide more precise fiber orientation estimates at the interface between tissues, thus yielding improved tractograms. In further works, such HYDI-based models may improve the quality of the obtained tractograms. Nevertheless, this investigation is beyond the scope of this study.

Diffusion MR microscopy has become a growing topic of interest in the diffusion MRI community, and models are constantly improving. Nowadays, the NODDI model has become very popular due to its easy implementation from the acquisition protocol to the analysis pipeline. Alternative models should be investigated in the future, in particular those probing further features like the mean axon diameter or the myelin water fraction. Because we took a special care to establish an acquisition protocol densely sampling the q and diffusion time spaces, the ActiveAx model could be investigated in the future using our HYDI diffusion data set. Its investigation is ongoing, but beyond the scope of this preliminary study. The latest improvements of the model now integrate a time dependence for the extra-axonal space that should allow to finely probe maps of the mean axon diameter within the hippocampus with fewer bias.

### Validation and comparison with alternative modalities

Ex vivo MRI is able to bridge the gap from the in vivo world to meso-scale configurations with a spatial resolution of 100–$200\ \upmu \hbox {m}$. In this study, we chose to limit our spatial resolution to 200–$300\ \upmu \hbox {m}$, respectively, for the anatomical and DW images to reach high *b* values (10,000$\ \hbox {s}/\hbox {mm}^{2}$) with a reasonable SNR to explore the properties of the tissue.

Novel optical methods are able to go down even further, to the microscopic scale. These methods include, in particular, optical coherence tomography (OCT), serial two-photon (STP) tomography, and 3D-polarized light imaging (3D-PLI). STP tomography (Ragan 2012) combines fluorescence imaging with two-photon microscopy, but requires the use of histochemical dyes to label the cells with type-specific fluorescent proteins, which is not the case of the two following methods, therefore, being sensitive only to the targeted cell populations. OCT (Magnain 2015) is a high-resolution (up to $1\,\upmu \hbox {m}$ in plane) optical technique analogous to ultrasound imaging as it measures the backscattered light of the sample, and is sensitive to differences in the refraction index in tissue. 3D-PLI (Axer 2011) gives the opportunity to observe the 3D orientation of the myelinated fibers without any staining procedure, thanks to the birefringence of the myelin sheath with an in-plane resolution of $1.3\,\upmu \hbox {m}$ and slices of $70\,\upmu \hbox {m}$. In contrast with other optical methods, whole human brain imaging is feasible, even if axons with a diameter at the range of the spatial resolution cannot be distinguished. This optical technique has already been applied to ex vivo human hippocampi by Zeineh et al. (2016), but the results have only been compared with in vivo DTI-based color-encoded maps. Despite their remarkable spatial resolution, optical methods also present inherent limitations compared with MRI. First, the sample has to be cut into slices for PLI and STP, and at least into blocks with a flat surface for OCT (Magnain 2014). Furthermore, contrary to dMRI, no real 3D acquisition is possible. Given the extremely large image size, supercomputing facilities are then required to precisely align the serial sections and produce three-dimensional reconstructions. Finally, diffusion MRI gives access to quantitative microstructural characteristics, like axonal density and diameter, which is not easily feasible using the novel optical methods described above.

Despite its own limitations, 3D-PLI can probably be considered as mature enough for the validations of diffusion MRI (Zeineh et al. 2016; Mollink et al. 2017). The human hippocampus sample scanned in the frame of this study is actually being analyzed using the 3D-PLI setup of our research partner. Tractography will be also performed from the 3D-PLI data and compared to the results of this work.

### Clinical prospects

This work has been done at an intermediate mesoscopic scale, between the micrometer obtained with optical methods and the millimeter obtained with in vivo MRI. It prefigures what could be achieved with ultra-high field clinical MRI. This is just the beginning of a new era of brain exploration. Advances in knowledge thanks to the ex vivo study, as well as microstructural models and hardware improvement, will allow, in fine, to consider a translational approach to reach the in vivo clinical routine.

Nowadays, there is no atlas of the human hippocampus connectivity and its microstructure at the mesoscopic scale. However, it would be of greatest value to clinical and cognitive neurosciences. Several tools are available to segment the hippocampus from MRI data [the object-based ROI module of the Anatomist software (http://www.brainvisa.info/index.html) in Boutet (2014), FIRST (FSL) or Freesurfer in Morey (2009)], and numerical atlases of the human hippocampus subfields have been established (Chupin 2009; Yushkevich 2010; Iglesias 2015). However, to our knowledge, no numerical atlas of their connectivity or their microstructure has been established. However, in most pathologies affecting the hippocampus, there is a need to better understand which subfields are affected, at what rate, and if the modifications induced by the pathology affect the neuronal cell bodies and/or their connections. For instance, Coras (2014) showed, in the case of hippocampal sclerosis (HS), that type 1 and 2 depicted different rates of cell loss with a more pronounced cell loss in CA3 and CA4 in type 1. Both kinds of HS samples depicted a contrast that did not allow the discrimination of the seven hippocampal layers contrary to normal samples, likely because of pathological shrinkage and fiber alteration.

Regarding structural modifications occurring with the normal process of aging, it is known that the hippocampus undergoes a particular volume decrease with age. MRI studies have suggested that, in typical aging, volume loss is more specific to CA1 and DG/CA3 subregions (Mueller and Weiner 2009). This volume loss is probably not due to neuronal cell loss (Riddle 2007), but rather to synapse loss (Burke and Barnes 2006), and occurs especially in the cortical inputs into the hippocampus such as the perforant pathway (Yassa et al. 2011). Wilson et al. (2006) also suggested that changes strengthen the auto-associative network of the CA3, amplifying the completion pattern (retrieval of previously stored information from a partial cue). The subject studied in this paper was an 87-year-old male. Regarding our results, that would mean that the outputs of the entorhinal cortex may be less significant than in a young hippocampus. As self-connections are excluded, there is no impact of the strengthening of the auto-associative network of CA3. Comparing these results to others obtained with a young hippocampus could highlight the reduction of the perforant path induced by the process of normal aging.

From a fundamental point of view, having access to a fine description of the hippocampal anatomy, including its subfields, its connectivity, and its microstructure, and being able to perform functional imaging using various memory tasks, opens the way to an improved functional neuroanatomy of the sensory, short-term, working, and long-term memories. Better understanding the neural networks driving these various cognitive processes might be useful to design, for instance, novel educational tools to improve the efficacy of young children to learn.

Finally, the protocol established for the human hippocampus could be generalized for the entire brain, and ensuing findings may help to push forward tractography algorithms. One of the limitations in tractography is that when a technique shows high sensitivity, i.e., a high rate of true positives, it most likely will show low specificity, i.e., a high rate of false positives (Thomas et al. 2014). Therefore, adding constraints arising from anatomical priors, like fine connectivity or microstructural characteristics, is intended to drastically reduce false positives creation.

## Conclusion

This study forms a proof-of-concept of how ultra-high field MRI with strong gradients can be applied to analyze hippocampal connectivity and microstructure ex vivo. It introduces a unique acquisition and segmentation protocol, and demonstrates that diffusion-weighted MRI offers a new opportunity to map the inner structural connectivity and microstructure of the human hippocampus, in good agreement with histology and current functional studies. The tractography and microstructure models highlight a higher connectivity and neurite density in the anterior hippocampus, whereas the intra-cellular volume fraction map reveals the laminar structure of the Ammon’s horn and could be used to improve segmentation protocols. In the future, these results could be of potential benefit to better correlate hippocampal atrophy, observed at low field in Alzheimer’s patients, with modifications of its inner connectivity and neurite density.

## Electronic supplementary material

Below is the link to the electronic supplementary material.

Supplementary material 1 (mp4 139142 KB)
